# Application of DNA barcoding for identification of freshwater carnivorous fish diets: Is number of prey items dependent on size class for *Micropterus salmoides*?

**DOI:** 10.1002/ece3.921

**Published:** 2013-12-23

**Authors:** Hyunbin Jo, Jeong-An Gim, Kwang-Seuk Jeong, Heui-Soo Kim, Gea-Jae Joo

**Affiliations:** 1 Department of Biological Sciences, Pusan National UniversityBusan, 609-735, South Korea; 2 Institute of Environmental Technology & Industry, Pusan National UniversityBusan, 609-735, South Korea

**Keywords:** Diet analysis, DNA barcoding, freshwater fish, predator–prey interaction, size class

## Abstract

Understanding predator–prey interactions is a major challenge in ecological studies. In particular, the accurate identification of prey is a fundamental requirement in elucidating food-web structure. This study took a molecular approach in determining the species identity of consumed prey items of a freshwater carnivorous fish (largemouth bass, *Micropterus salmoides*), according to their size class. Thirty randomly selected gut samples were categorized into three size classes, based on the total length of the bass. Using the universal primer for the mtDNA cytochrome oxidase I (COI) region, polymerase chain reaction (PCR) amplification was performed on unidentified gut contents and then sequenced after cloning. Two gut samples were completely empty, and DNA materials from 27 of 28 gut samples were successfully amplified by PCR (success rate: 96.4%). Sequence database navigation yielded a total of 308 clones, containing DNA from 26 prey items. They comprised four phyla, including seven classes, 12 orders, and 12 families based on BLAST and BOLD database searches. The results indicate that largemouth bass show selective preferences in prey item consumption as they mature. These results corroborate a hypothesis, presence of ontogenetic diet shift, derived through other methodological approaches. Despite the practical limitations inherent in DNA barcoding analysis, high-resolution (i.e., species level) identification was possible, and the predation patterns of predators of different sizes were identifiable. The utilization of this method is strongly recommended for determining specific predator–prey relationships in complex freshwater ecosystems.

## Introduction

Understanding predator–prey interactions is one of the major challenges in ecological studies (Carreon-Martinez and Heath 2010). Analysis of prey selection and food web is largely dependent on the resolution of food-web component identification; therefore, the better we can accurately identify prey species, the better we can understand the system (Hardy et al. 2010; Carreon-Martinez et al. 2011). Previous diet studies have revealed important information regarding predator–prey interactions; however, the significance of the results of these studies has been limited to some extent due to the analytical methodology employed. Gut or fecal analysis is a fundamental step in the determination of predator–prey relationships (Kuch et al. 2002), and visual inspection has conventionally been used for the analysis of gut contents or fecal materials. However, most studies based on visual inspection have the following disadvantages: ambiguous prey specimen identification due to extensive digestion, the presence of unidentified partial tissues, a lack of expert knowledge for avoiding identification failure, and low-level identification resolution (higher than family or order level). In addition, visual inspection is hampered in the case of smaller-sized predators.

The emergence of several new techniques, such as fatty acid signatures, stable isotopes, and DNA methods, may overcome these limitations. Fatty acid or stable isotope analyses can provide a substantive picture of energy and material flow through the food web; however, they are not appropriate for revealing predator–prey specific interactions in complex ecosystems (Hardy et al. 2010). Applying DNA techniques to diet identification has recently increased identification resolution, particularly in marine ecosystems (Blankenship and Yayanos 2005; Durbin et al. 2008, 2012; Nejstgaard et al. 2008; Riemann et al. 2010; Cleary et al. 2012). However, very few studies have used DNA barcoding for dietary analysis in complex freshwater ecosystems (Garros et al. 2008; Corse et al. 2010; Carreon-Martinez et al. 2011) and have recognized this technique as a promising tool in studying trophic interactions (Andrew et al. 2013).

Carnivorous species play important roles in the determination of predator–prey interactions (Fritts and Rodda 1998). The largemouth bass (*Micropterus salmoides*) is one of the most common carnivorous freshwater fish species in the world (Welcomme 1992), and its impact on food-web structure and function has been widely investigated (Blanco et al. 2003; Weyl et al. 2010; Ellender et al. 2011). Largemouth bass less than 80 mm in body length prey mainly on invertebrates, and as they grow their diet shifts gradually to fish and crayfish (Olson 1996; García-Berthou 2002; Post 2003; Jang et al. 2006; Yasuno et al. 2012). Thus, each growth stage may play a functionally different role in freshwater ecosystems, and largemouth bass can affect a wide range of prey species as well as prey size (García-Berthou 2002; Nakazawa et al. 2007). However, despite the fact that these studies have discovered such relationships between the largemouth bass and its prey, our current knowledge may be improved by increasing prey item resolution, in order to investigate “complex” networks of predators and prey. The use of DNA barcoding may provide an opportunity of improving our present understanding of predator–prey relationships.

The objectives of this study were to examine the pattern of diet selection of predatory fish in a large freshwater wetland using the DNA barcoding method, based on the universal primer region of cytochrome oxidase I (COI). DNA barcoding can enable the characterization and monitoring of biodiversity in a target ecosystem (Hebert et al. 2003) and can make it possible to identify prey items at the species level. We aimed to evaluate (1) the pattern of prey item selection in accordance with largemouth bass size classes and (2) the applicability and effectiveness of DNA barcoding in prey identification. Our results are discussed in relation to previous studies concerning the effectiveness of DNA barcoding in food-web analysis.

## Materials and Methods

### Study site and fish collection

The study site was in the Upo Wetlands (35°31′34.51′′N, 128°22′34.94′′E). This large wetland is one of the most important freshwater ecosystems in South Korea and has been designated a Ramsar Conservation Site for its high biodiversity and for the protection of its water-bird habitat (Kim et al. 2004; Do et al. 2007; Jo et al. 2011). Non-native largemouth bass were first discovered at Upo in 1996, and this ecosystem might have suffered because of the introduced species.

Largemouth bass were caught with cast nets (7 × 7 mm mesh size) and scoop nets (5 × 5 mm mesh size) at the study site. We conducted sampling in 2010, 2012, and 2013. Basic morphological parameters (total length, body length, and biomass) of collected largemouth bass were measured immediately after capture, and their guts were eviscerated for gut content analysis. To avoid contamination with foreign-derived DNA, we clenched the lower esophageal part using forceps and eviscerated the guts using medical scissors rinsed in 98% methanol. Gut samples were preserved in 99% ethanol and stored at room temperature before analysis. From stored samples (total ca. 180 samples), we randomly selected 30 guts for the identification of ingested prey items by DNA cloning. The gut samples were categorized into three groups according to size class based on total length (TL) as follows: size class I (TL < 100 mm [*n* = 10]); size class II (TL 100–199 mm [*n* = 10]); size class III (TL ≥ 200 mm [*n* = 10]) (Table 1).

**Table 1:** Sample and polymerase chain reaction (PCR) amplification information.

No.	ID	TL (mm)	BL (mm)	WT (g)	Date	PCR success	No. of time PCR	No. clones
1	B-3	31	24	0.3	2012.6.13	Success	1	10
2	A-7-1	32	25	0.3	2012.6.13	Success	1	10
3	B-5	34	28	0.4	2012.6.13	Success	1	8
4	B-7	35	27	0.4	2012.6.13	Success	1	10
5	A-7-3	36	30	0.3	2012.6.13	Success	1	10
6	B-4	36	29	0.3	2012.6.13	Success	1	10
7	A-7-2	38	30	0.3	2012.6.13	Success	1	10
8	B-6	39	31	0.3	2012.6.13	Success	2	10
9	B-2	40	32	0.5	2012.6.13	Success	1	7
10	B-1	43	34	0.3	2012.6.13	Failed	2	
				**Size class I**			**Subtotal**	**85**
11	A-3-1	103	86	11.7	2012.3.30	Success	1	10
12	B-23	140	116	36.1	2013.4.18	Success	1	10
13	B-22	143	115	34.9	2013.4.18	Success	1	11
14	A-2-1	159	132	36.0	2012.3.30	Success	1	11
15	B-8	162	140	58.1	2010.10.21	Success	2	10
16	A-4-1	163	138	46.3	2012.6.13	Success	1	32
17	B-21	172	144	63.1	2013.4.18	Success	1	11
18	A-6-1	177	146	63.5	2012.6.13	Success	2	11
19	B-13	196	167	79.8	2010.5.6	Success	1	11
20	B-9	198	205	152.0	2010.5.6	Success	1	25
				**Size class II**			**Subtotal**	**142**
21	A-1-1	216	185	109.8	2012.3.30	Success	1	10
22	B-20	235	195	150.5	2010.5.6	Success	1	11
23	B-15	240	196	150.8	2010.5.6	Success	2	10
24	A-5-1	250	215	164.3	2012.6.13	Success	1	10
25	B-19	293	246	280.1	2010.5.8	Success	1	10
26	B-24	330	282	578.1	2010.8.30	Empty stomach		
27	B-21 (B)	331	285	572.0	2010.8.31	Empty stomach		
28	B-11	354	290	586.8	2012.9.7	Success	1	10
29	B-17	357	314	586.8	2010.5.6	Success	2	10
30	B-16	367	324	678.5	2010.5.6	Success	1	10
				**Size class III**			**Subtotal**	**81**
							**Total**	**308**

BL, body length; TL, total length; WT, weight.

### DNA extraction, PCR amplification, and cloning

Gut contents were extracted and kept separately in a fresh state. They were then rinsed in autoclaved water to avoid contamination with foreign-derived and self-DNA. Ethanol was completely volatilized from the samples preceding the DNA extraction process. The samples were then frozen with liquid nitrogen and homogenized. Genomic DNA from each of the gut samples was isolated using the LaboPass Tissue Miniprep Kit (Cosmogenetech, Seoul, Korea) according to the manufacturer's manuals.

Polymerase chain reaction (PCR) amplification was performed using G-Taq DNA polymerase (Cosmogenetech) with 10 *μ*g of genomic DNA and 0.1 *μ*mol/L primers in a final volume of 20 *μ*L. The COI region was amplified with LCO1490: (5′-GGTCAACAAATCATAAAGATATTGG-3′) and HCO2198: (5′-TAAACTTCAGGGTGACCAAAAAATCA-3′; Folmer et al. 1994). The PCR thermal regime consisted of one cycle of 1 min at 94°C; five cycles of 1 min at 94°C, 1.5 min at 45°C, and 1.5 min at 72°C; 35 cycles of 1 min at 94°C, 1.5 min at 50°C, and 1 min at 72°C; and a final cycle of 5 min at 72°C in a Mastercycler (Eppendorf, Hamburg, Germany). PCR products were separated by 1.5% agarose gels. If the samples were not amplified satisfactorily in the first attempt, re-amplification was performed using 1 *μ*L of the first PCR products, following the previous experimental protocol. After purification using a Labopass Gel Extraction Kit (Cosmogenetech), cloning was carried out using the pGEM-T easy vector (Promega, Madison, WI). Cloned plasmid DNA was isolated according to the alkaline-lysis method using a Labopass Plasmid Miniprep Kit (Cosmogenetech). Individually isolated plasmid DNA was then digested using the restriction enzyme *Eco*RI to confirm insertion. Ten or 11 positive clones for each sample were analyzed to species-specific sequences on SP6 primers using an automated 3730 DNA analyzer (Applied Biosystems, Foster City, CA), except for samples B-2 and B-5, which provided only seven and eight positive clones, respectively (Table 1).

An additional clone selection was undertaken to ascertain the number of identified prey species. The gut samples with the largest number of prey items were selected, and extra clones were sequenced following the aforementioned process.

### DNA sequence analysis

Sequence alignment was performed using Clustal W 2.0 (Larkin et al. 2007). A BLASTn search was performed to find obtained sequences with the best hits. Ten sequences of the top hits from GenBank and BOLD systems, in addition to two or three out-groups from the nearest families, were downloaded. The degree of similarity between obtained sequences was assessed using the neighbor-joining (NJ) algorithm (Saitou and Nei 1987) as implemented in MEGA 5.0 (Tamura et al. 2011). The degree of information redundancy in fragments compared using NJ was assessed by bootstrap resampling of 1000 pseudoreplicate data sets (Felsenstein 1985).

We adopted two criteria to establish accurate species identification: (1) that there was identification of an operational taxonomic unit (OTU) with a ≥98% identity with a known species (a 2% difference between an OTU and a known species may be caused by intraspecific variation or PCR and sequencing errors (Jarman et al. 2004; Clare et al. 2009)) and (2) that the phylogenetic tree constructed returned a reasonable clustering of the sequences of OTU and known species. If a sequence comparison between an OTU and a known species was ≥98% similar, the recognized species information of the OTU was accepted. If a sequence comparison between an OTU and a known species was <98% similar, the OTU was recognized at a higher classification level (i.e., genus, family, etc.). Although species identity was accepted at a similarity of ≥98%, if the phylogenetic tree clustering was not conclusive (i.e., the OTU was not reasonably grouped with any known species’ sequence), the OTU was identified at a higher classification level. For example, if a sequence matched members of a known genus but could not be unequivocally identified due to incomplete taxonomic coverage in the reference database (<98% identity), and was clustered to a clade at the genus level, the sequence was considered to be a genus-level identification. If it was clustered to a clade at the family or order level, the sequence was considered to be a family-or order-level identification.

## Results

### Gut content analysis based on DNA barcoding

DNA barcoding analysis returned a list of consumed prey items at a reasonably high resolution. From the 30 gut samples, two samples were completely empty. DNA materials from 27 of 28 gut samples were successfully amplified by PCR (96.4% success rate; Table 1). We sequenced 308 clones of prey items and obtained robust 658-bp sequence data; the clones contained DNA from 26 prey items. Among these, 15 prey items were clearly identified at the species level (57.7% of 26 prey items). The identification accuracy of the remaining 11 prey items (42.3%) ranged between 83% and 97%, and so could only be identified to the genus level or higher.

Following the adoption of the identification criteria described in the Methods, we determined that prey items were found from four phyla, including seven classes, 12 orders, and 12 families, based on BLAST and BOLD database searches and phylogenetic tree construction (Table 2, Fig. S1). Insecta comprised the largest proportion (13 OTUs, 50.00%) followed by Actinopterygii (five OTUs, 19.23%), and Malacostraca (three OTUs, 11.54%). The largest number of OTUs was from the Chironomidae, consisting of nine different sequences (at species, genus, or family level). Most prey items possessed hard body parts (e.g., bones, exoskeletons, teeth), but several species that did not have hard bodies, including *Metaphire hilgendorfi* and *Hydra oligactis*, were identified with >99% accuracy. For more detailed identification results, refer to the supplementary results (Fig. S1).

**Table 2:** Animal taxa identified in the diet of largemouth bass, based on sequence variation in the cytochrome oxidase I region using stomach contents. Raw sequences and phylogenetic trees of each species are given in Table S1 and Figure S1.

	Sample number along the total length^1^																																	
Prey organisms	1	2	3	4	5	6	7	8	9	10	11	12	13	14	15	16	17	18	19	20	21	22	23	24	25	26	27	28	29	30	Total	Identity (%)	GenBank accession	Level of identification
**Animalia**																																		
Phylum Annelida																																		
Class Oligochaeta																																		
Order Haplotaxida																																		
Family Megascolecidae																																		
*Metaphire hilgendorfi*														3				3													6	99	AB542630.1	Species
Phylum Arthropoda																																		
Class Malacostraca																																		
Order Isopoda																																		
Family Asellidae																																		
*Asellus sp. 1*												1	1				1		1	3	10	2							9		28	89	AY531829.1	Genus
*Asellus sp. 2*																							1						1		2	83	DQ144785.1	Genus
Order Decapoda																																		
Family Palaemonidae																																		
*Macrobrachium nipponense*																1			2						7						10	100	JN874519.1	Species
Class Branchiopoda																																		
Order Diplostraca																																		
Family Daphniidae																																		
*Daphnia sp*.											2																				2	98	EF375867.1	Genus
Class Maxillopoda																																		
Order Cyclopoida																																		
Family Cyclopidae																																		
*Cyclops sp. 1*											1																				1	97	KC627290	Genus
*Cyclops sp. 2*											4																				4	96	KC627290	Genus
Class Insecta																																		
Order Odonata																																		
Family Coenagrionidae																																		
*Paracercion calamorum*								8			1																				9	100	AB708522.1	Species
*Paracercion hieroglyphicum*								2	3																						5	99	AB708524.1	Species
* Paracercion sp*.									1																						1	96	AB708534.1	Genus
Order Diptera																																		
Family Chironomidae																																		
*Chironomus kiiensis*	6																														6	99	KC407765.1	Species
*Chironomus nipponensis*							3																								3	99	JN887051.1	Species
*Chironomus plumosus*				2								3			1					1			1								8	99	KC407771.1	Species
*Glyptotendipes tokunagai*				8								1	5			1	8		1	1				6							31	99	JQ350718.1	Species
*Dicrotendipes nervosus*	2	1														4															7	99	JF412128.1	Species
*Polypedilum cultellatum*																	1														1	99	JF412156.1	Species
*Chironomus sp.1*	2	9	6		10	10	7				2					1		1													48	96	JF412075.1	Genus
*Chironomus sp.2*			2																												2	99	JF412065.1	Genus
Chironomidae																1															1	90	AY752674.1	Family
Order Ephemeroptera																																		
Family Caenidae																																		
Caendidae									3																						3	82	JQ662051.1	Family
Phylum Cnidaria																																		
Class Hydrozoa																																		
Order Anthomedusae																																		
Family Hydridae																																		
*Hydra oligactis*														6																	6	99	GU722868.1	Species
Phylum Chordata																																		
Class Actinopterygii																																		
Order Cypriniformes																																		
Family Cyprinidae																																		
*Hemibarbus labeo*														1		1				5					2					10	19	99	HQ536371.1	Species
*Opsariichthys uncirostris amurensis*														1																	1	99	HQ536421.1	Species
Cyprinidae																1															1	98	HQ536348.1	Family
Order Siluriformes																																		
Family Bagridae																																		
*Tachysurus fulvidraco*																			7						1			10			18	99	HM641815.1	Species
Order Perciformes																																		
Family Centrarchidae																																		
*Micropterus salmoides*												4	5		9			7				9	8	4							46	100	DQ536425.1	Species
Number of colony sequences	10	10	8	10	10	10	10	10	7		10	10	11	11	10	10	11	11	11	10	10	11	10	10	10			10	10	10	271			
Number of diet species	3	2	2	2	1	1	2	2	3		5	4	3	4	2	7	3	3	4	4	1	2	3	2	3			1	2	1	26			

1 Information of sample number (sample number: total length); 1: 31 mm/2: 32/3: 34/4: 35/5: 36/6: 36/7: 38/8: 39/9: 40/10: 43/11:103/12: 140/13: 143/14: 159/15: 162/16: 163/17: 172/18: 177/19: 196/20: 198/21: 216/22: 235/23: 240/24: 250/25: 293/26: 330/27: 331/28: 354/29: 357/30: 367.

The samples A-4-1 and B-9 contained relatively large numbers of prey items, and we sequenced an extra 22 and 15 clones, respectively, based on a random selection of clones. This sequencing did not return any increase in prey items.

### Prey selection with respect to predator size class

**Figure 1:**
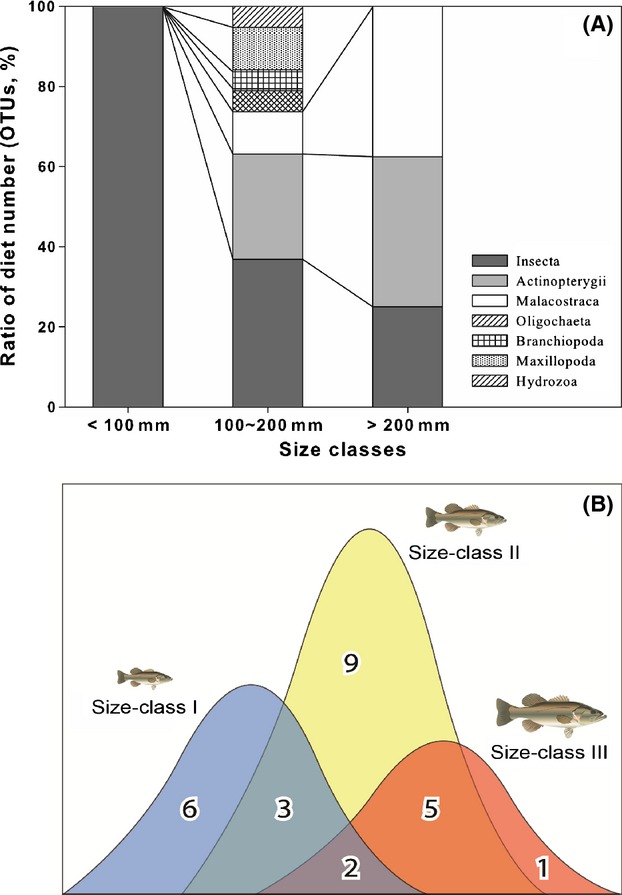
(A) Proportion of operational taxonomic units (OTUs; %) in each predator size class and (B) nondimensional Venn diagram showing number of OTUs by predator size class.

Largemouth bass showed different preferences in consumed prey items as they grew. The number of prey items was high when a bass's TL was >100 mm. However, the number of prey items declined when the bass were >200 mm in TL. Size class II (100–199 mm TL) utilized the largest number of prey items (Fig. 1A). A clearly distinguishable pattern of prey item composition was observed: Small bass (size class I) consumed only class Insecta, while bass in size class II consumed seven classes, including Insecta, Actinopterygii, and Malacostraca. The largest bass (size class III) relied on a narrow prey spectrum (three classes). Figure 1B illustrates the prey item consumption patterns of the three size classes. A large proportion of the prey items found in the largest bass (i.e., size class III) were also found in the other size classes, whereas the bass of intermediate size (size class II) targeted largely different prey items from the small and large bass.

## Discussion

### The pattern of prey selection in largemouth bass size classes

Carnivorous fish that undergo large changes in body size typically show a remarkable shift in resource use along the body length gradient (Post 2003). The timing of diet shift is particularly important for predator and prey species for which resource use, growth rate, and predation risk are strongly related to body size (Olson 1996). The data presented in Figure 1A suggest that when bass are small (<100 mm, class I), they have a small mouth and limited swimming abilities; consequently, they cannot eat large prey, or prey with well-developed swimming abilities. Therefore, small bass are restricted in their prey selection. However, when they grow to >100 mm, they dramatically increase their prey species range because they are able to swim better and have mouths large enough to swallow larger prey species (Persson and Greenberg 1990). Figure 1B shows that predators in size class III consumed six prey species not found in smaller predators (class I); however, most prey species overlapped with class II, and only one species was solely predated by the one in size class III. Largemouth bass may need to balance efficiency in prey consumption with body size maintenance, which could explain why the largest individuals consumed a relatively small number of prey items, fewer than that of size class I.

These results suggest possible approaches to the management of largemouth bass populations in order to minimize their impact on the native species they prey on. If control measures are focused on large-and medium-sized largemouth bass, then more effective conservation is possible. Elimination of individual largemouth bass should be based on the observed patterns of population dynamics, and juvenile removal is fundamental to population management. Nevertheless, DNA barcoding provides not only insights into dietary shift analysis for estimating the impact of predator size classes on prey populations, but also provides a tool for the effective management of an invasive species. Additional experimental studies, based on the approach taken in the present study, are necessary to develop a firm management strategy. This is particularly important for ecosystems with a high biodiversity, and complex food-web structures, such as the Upo Wetlands.

### The significance of DNA barcoding in prey selection analysis and its potential limitations

Previous studies of dietary shifting have mainly focused on the timing of changes in diet (Olson 1996; García-Berthou 2002; Post 2003; Jang et al. 2006; Yasuno et al. 2012) and were relatively constrained in their ability to identify prey items to species level due to a lack of specific prey data. Species-level identification allows us to investigate the impact of largemouth bass populations at a fundamental level so that species-specific interactions can be identified. This approach confers two advantages over other methods: (1) The range of predator age classes open to study is widened; and (2) prey identification to the species level is possible. In the past, small fish, including juvenile fish, could not be studied because their guts were too small to be examined. Samples obtained from such fish had to be analyzed with great expertise and knowledge. Therefore, despite the importance of assessing prey in terms of predator size (Huss et al. 2008), conventional analyses often ignored juveniles. However, if the surgical evisceration of the gut is possible (either for juveniles or adults), prey analysis can be successfully carried out (see Table 2) so that a detailed understanding of the effects of predator populations on biodiversity can be based on effective species-wise differentiation (Pompanon et al. 2012). The second advantage of DNA barcoding is the high resolution of prey identification. Visual inspection is often impeded by the incomplete nature of the prey specimen, and digestion degrades prey specimens, resulting in identification failure. This problem can be overcome by the DNA barcoding process. High-resolution characterization of food-web structure is possible, and detailed remedial strategies for species management (either predator or prey) are attainable. Of course, the impact of predators on prey species should also be investigated quantitatively, as well as through the qualitative identification of prey species by DNA barcoding.

Notwithstanding the importance of DNA analysis, problems with DNA barcoding using universal primers with cloning do exist. Sensitive PCR mastercycler and universal primers can cause two problems: (1) detecting secondary predation; and (2) self-DNA contamination. DNA prey analysis can be very sensitive to secondary predation (Sheppard and Harwood 2005; O'Rorke et al. 2012). In the present study, we used a specialized primer set (COI), which is targeted at the cells of prey species; therefore, it is possible that the analysis may have included instances of secondary predation. Secondly, self-DNA contamination is a common problem in DNA barcoding research. Because prey samples were collected from the predators’ guts, there is a very high probability that predator DNA was included in the prey samples. However, the problem of self-DNA contamination can be resolved by blocking the detection of predator DNA with ligase and a blocking oliogonucleotide (Cleary et al. 2012; Craig et al. 2013).

### Quantifying selected prey items

Direct sequencing or cloning with PCR products provides only “presence or absence” data. Therefore, the objectives of prey selection analysis should be carefully considered when using the DNA barcoding approach. The quantification problem can be partly overcome by utilizing the intermediate products of the process. Cloning includes several experimental stages, and counting the number of cloned colonies is possible (see Table 2). Although simply counting the number of colonies in a sample does not provide a complete and accurate picture of prey abundance (there is a difference between the number of prey items and the number of clone samples), it can be used to calculate the proportion of different clone sequences (i.e., prey species). If a relationship between colony counts and the biomass of a prey species is found, this information may be useful for the quantification of prey items. The number of clones (i.e., 10 or 11 clones) adopted in the current study was insufficient for prey item quantification. When investigation of whole clones is available, researchers can quantify prey items using indexing systems such as the Index of Relative Importance or the Costello method (Pinkas 1971; Amundsen et al. 1996). Next-generation sequencing (NGS), which is still relatively expensive and difficult to carry out, may provide an opportunity to overcome this problem, and the preparation of a large database of sequence inventories will encourage the approach of studying dietary analysis based on greater identification resolution to species level and the relative quantification of the prey items.

## Conclusion

In this study, we identified the prey species consumed by a predator (largemouth bass) in a freshwater ecosystem. High-resolution (i.e., species level) identification was possible, and smaller-sized predators (i.e., juveniles) were successfully included. Despite the limitations of DNA barcoding analysis, utilization of this method is strongly recommended for determining specific predator–prey relationships in complex freshwater ecosystems. The fruitful investigation of species-level interactions between predators and prey will lead to more precise food-web characterizations, based on wider ranges of prey species, and to more accurate evolutionary and ecological food-web modeling.

## Data Accessibility

Raw sequences of prey species and their BLASTn search results are available as supplementary materials, Table S1 (the attached MS Excel file).

## Supporting Information

Figure S1 Alignment of cytochrome oxidase I region for phylogeny tree by MEGA5.

Table S1 Raw sequences data and BLASTn search of each individual species used in this study.
